# Supplementary material for: Usefulness of Machine Learning-Based Gut Microbiome Analysis for Identifying Patients with Irritable Bowels Syndrome
Source: J Clin Med. 2020 Jul 27;9(8):2403. doi: 10.3390/jcm9082403 (PMC7464323; doi:10.3390/jcm9082403)
Supplement: Supplementary file 1 [file jcm-09-02403-s001.zip › Revise Suppl Figures & Table & data/Supplementary Table 1.docx]

**Supplementary Table 1.** Taxa associated with IBS. Each OTUs was assigned by BLAST search.

| OTU | lasso score | NCBI taxonomy | E value | Identity | Accession |
| --- | --- | --- | --- | --- | --- |
| OTU6724 | 1.32 | Halomonas hamiltonii | 2e-165 | 99.69 | NR_115089.1 |
| OTU18561 | 0.54 | Klebsiella quasivariicola | 4e-178 | 99.42 | CP022823.1 |
| OTU2926 | 0.14 | Fusicatenibacter saccharivorans | 2e-171 | 97.99 | NR_114326.1 |
| OTU17578 | 0.13 | Blautia stercoris | 1e-163 | 100 | NR_117867.1 |
| OTU14480 | 0.13 | Ihubacter massiliensis | 2e-126 | 89.53 | NR_144749.1 |
| OTU16925 | 0.12 | Eubacterium oxidoreducens | 7e-121 | 89.2 | NR_104737.1 |
| OTU19176 | 0.10 | Prevotella copri | 2e-165 | 96.35 | NR_040877.1 |
| OTU25404 | 0.08 | Bacteroides coprocola | 5e-172 | 97.74 | NR_041278.1 |
| OTU216 | 0.07 | Bacteroides thetaiotaomicron | 8e-160 | 96.01 | AE015928.1 |
| OTU18732 | 0.07 | Streptococcus lactarius | 7e-161 | 97.34 | NR_117425.1 |
| OTU28261 | 0.05 | Oscillibacter valericigenes | 5e-157 | 94.49 | NR_074793.2 |
| OTU17293 | 0.04 | Parabacteroides goldsteinii | 2e-170 | 97.99 | EU136697.1 |
| OTU27426 | 0.02 | Faecalibacterium prausnitzii | 2e-146 | 94.94 | NR_028961.1 |
| OTU29246 | -0.01 | Fusicatenibacter saccharivorans | 1e-148 | 94.02 | NR_114326.1 |
| OTU22960 | -0.03 | [Eubacterium] hallii | 3e-149 | 93.09 | NR_118673.1 |
| OTU24792 | -0.05 | Collinsella aerofaciens | 5e-157 | 96.2 | NR_113316.1 |
| OTU4167 | -0.05 | Eubacterium rectale | 8e-155 | 93.97 | FP929042.1 |
| OTU24770 | -0.08 | Bifidobacterium adolescentis | 3e-164 | 97.13 | AP009256.1 |
| OTU31478 | -0.08 | Faecalibacterium prausnitzii | 7e-151 | 95.56 | NR_028961.1 |
| OTU16016 | -0.09 | Emergencia timonensis | 5e-137 | 91.16 | NR_144737.1 |
| OTU21851 | -0.09 | Bacteroides massiliensis | 2e-155 | 94.97 | NR_042745.1 |
| OTU8331 | -0.09 | Anaeromassilibacillus senegalensis | 2e-146 | 93.75 | NR_144727.1 |
| OTU734 | -0.10 | Blautia obeum | 3e-154 | 96.96 | NR_118692.1 |
| OTU32554 | -0.11 | Collinsella aerofaciens | 2e-165 | 97.67 | NR_113316.1 |
| OTU18054 | -0.12 | Cuneatibacter caecimuris | 2e-137 | 91.26 | NR_144608.1 |
| OTU12852 | -0.12 | Ihubacter massiliensis | 2e-126 | 89.53 | NR_144749.1 |
| OTU17109 | -0.12 | Bacteroides vulgatus | 2e-180 | 99.15 | NR_074515.1 |
| OTU4423 | -0.14 | Blautia obeum | 2e-162 | 98.48 | NR_118692.1 |
| OTU21673 | -0.15 | Bacteroides xylanisolvens | 4e-163 | 97.36 | NR_112947.1 |
| OTU10015 | -0.15 | Bacteroides massiliensis | 2e-180 | 99.43 | NR_042745.1 |
| OTU14692 | -0.17 | Bacteroides vulgatus | 3e-160 | 96.01 | CP000139.1 |
| OTU29988 | -0.17 | Coprobacter fastidiosus | 4e-178 | 98.87 | NR_118316.1 |
| OTU18195 | -0.20 | Faecalibacterium prausnitzii | 2e-142 | 94.08 | NR_028961.1 |
| OTU21277 | -0.21 | Dorea longicatena | 7e-126 | 90.08 | LC037228.1 |
| OTU19250 | -0.24 | Parabacteroides distasonis | 9e-170 | 98.26 | CP000140.1 |
| OTU8107 | -0.27 | Faecalibacterium prausnitzii | 3e-160 | 96.79 | NR_028961.1 |
| OTU20544 | -0.29 | Fusicatenibacter saccharivorans | 2e-155 | 95.16 | NR_114326.1 |
| OTU10359 | -0.38 | Bacteroides vulgatus | 5e-172 | 97.74 | CP000139.1 |
| OTU20913 | -0.42 | Parabacteroides distasonis | 2e-156 | 95.21 | NR_074376.1 |
