# Supplementary figures and images for: Usefulness of Machine Learning-Based Gut Microbiome Analysis for Identifying Patients with Irritable Bowels Syndrome
Source: J Clin Med. 2020 Jul 27;9(8):2403. doi: 10.3390/jcm9082403 (PMC7464323; doi:10.3390/jcm9082403)

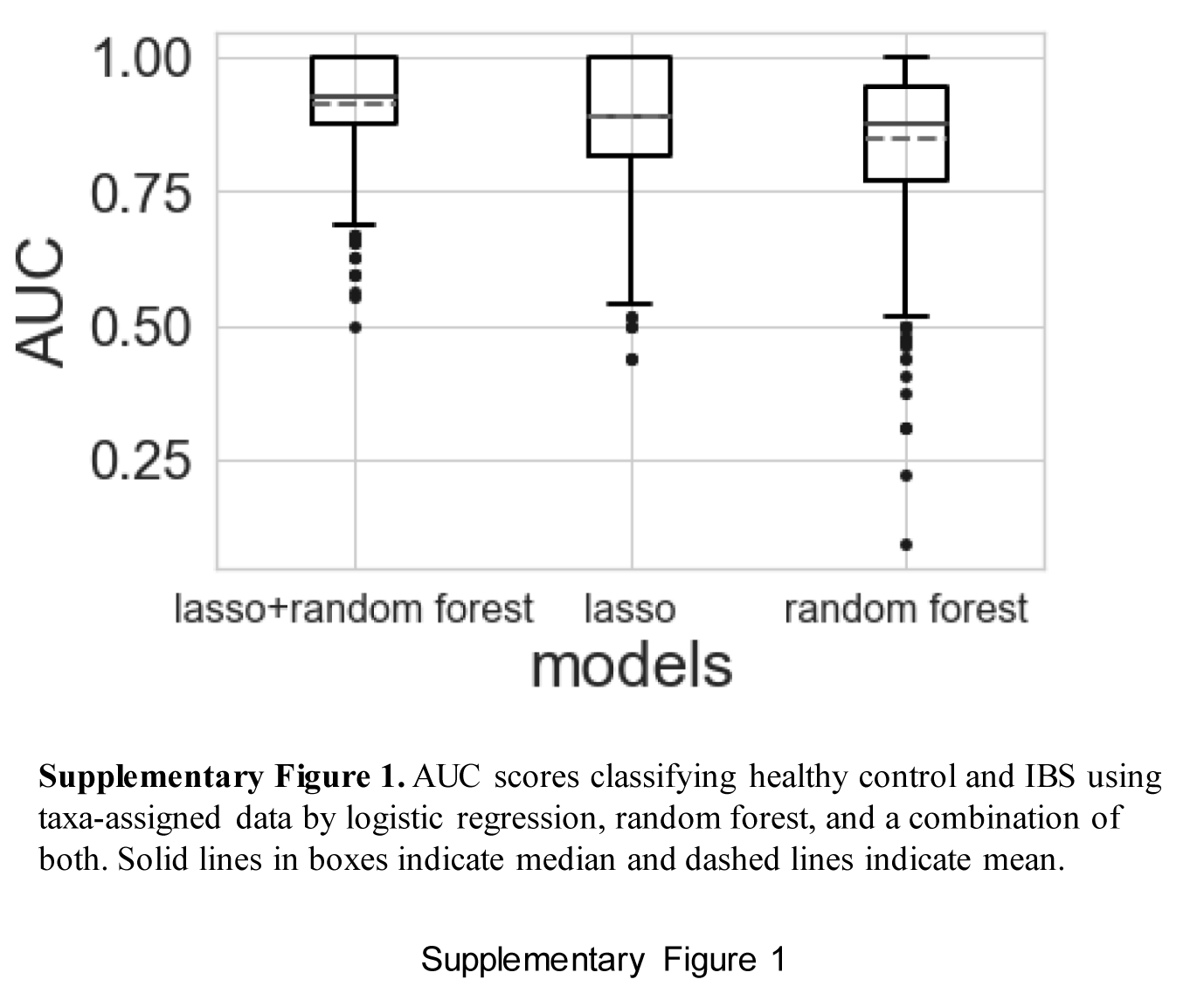

Supplement: Supplementary file 1 [file jcm-09-02403-s001.zip › Revise Suppl Figures & Table & data/Supplementary Figure 1 revise.tif]

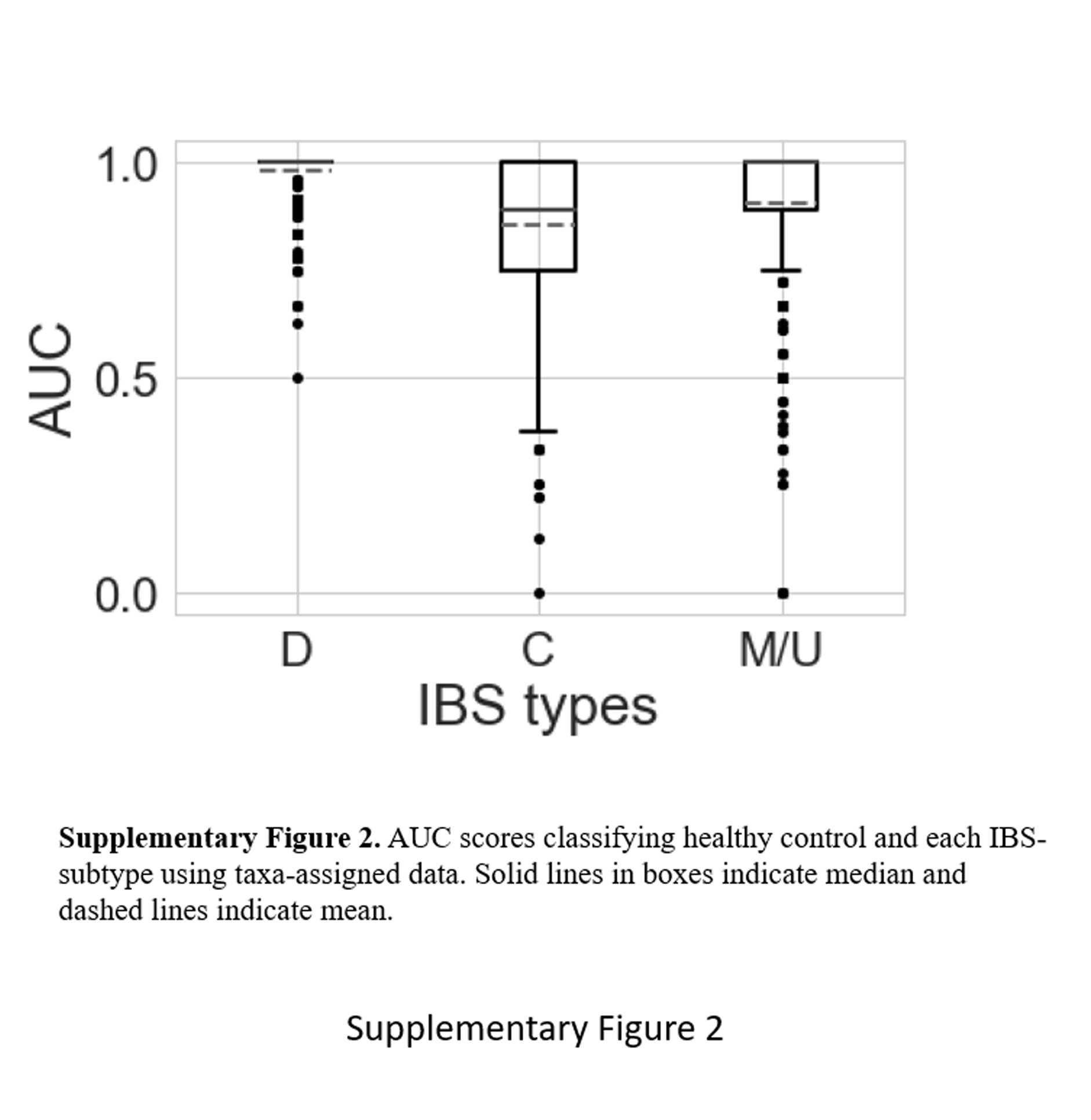

Supplement: Supplementary file 1 [file jcm-09-02403-s001.zip › Revise Suppl Figures & Table & data/Supplementary Figure 2.tif]
